# Inhibition of Enzyme and Bacteria Activities in Diabetic Ulcer-like Scenarios via WAAPV-Loaded Electrospun Fibers

**DOI:** 10.3390/pharmaceutics16070911

**Published:** 2024-07-08

**Authors:** Ana R. M. Ribeiro, Catarina S. Miranda, Ana Francisca G. Silva, Filipa D. P. Mendes, Beatriz M. Silva, Bruna A. S. Oliveira, Eduardo D. Paiva, Sónia P. Gonçalves, Sílvia M. M. A. Pereira-Lima, Susana P. G. Costa, Helena P. Felgueiras

**Affiliations:** 1 Centre for Textile Science and Technology (2C2T), University of Minho, Campus of Azurém, 4800-058 Guimarães, Portugal; anarribeiro@2c2t.uminho.pt (A.R.M.R.); catarina.miranda@2c2t.uminho.pt (C.S.M.); pg50248@alunos.uminho.pt (B.M.S.); pg49861@alunos.uminho.pt (B.A.S.O.); pg53798@alunos.uminho.pt (E.D.P.); sonia.pires@2c2t.uminho.pt (S.P.G.); 2 Centre of Chemistry (CQ), University of Minho, Campus of Gualtar, 4710-057 Braga, Portugal; id10809@alunos.uminho.pt (A.F.G.S.); silviap@quimica.uminho.pt (S.M.M.A.P.-L.); spc@quimica.uminho.pt (S.P.G.C.)

**Keywords:** polycaprolactone/polyethylene glycol fibers, electrospinning, co-adjuvant antibacterial action, proteolytic enzyme inhibition

## Abstract

In diabetic ulcers, an increased secretion of human neutrophil elastase (HNE) and bacterial infections play crucial roles in hindering healing. Considering that, the present study proposed the development of multi-action polycaprolactone (PCL)/polyethylene glycol (PEG) electrospun fibers incorporating elastase-targeting peptides, AAPV and WAAPV, via blending. Characterization confirmed WAAPV’s efficacy in regulating proteolytic enzymes by inhibiting HNE. The engineered fibers, particularly those containing PEG, exhibited optimal wettability but an accelerated degradation that was mitigated with the peptide’s inclusion, thus promoting a sustained peptide release over 24 h. Peptide loading was verified indirectly through thermal stability and hydration capacity studies (hydrophobic bonding between PCL and WAAPV and hydrophilic affinities between PCL/PEG and AAPV) and determined at ≈51.1 µg/cm^2^ and ≈46.0 µg/cm^2^ for AAPV and ≈48.5 µg/cm^2^ and ≈51.3 µg/cm^2^ for WAAPV, respectively, for PCL and PCL/PEG. Both AAPV and WAAPV effectively inhibited HNE, with PEG potentially enhancing this effect by interacting with the peptides and generating detectable peptide–PEG complexes (≈10% inhibition with PCL + peptide fibers after 6 h of incubation, and ≈20% with PCL/PEG + peptide fibers after 4 h incubation). Peptide-loaded fibers demonstrated antibacterial efficacy against *Staphylococcus aureus* (up to ≈78% inhibition) and *Escherichia coli* (up to ≈66% inhibition), with peak effectiveness observed after 4 and 2 h of incubation, respectively. This study provides initial insights into the WAAPV’s potential for inhibiting HNE and bacteria activities, showing promise for applications in diabetic ulcer management.

## 1. Introduction

Diabetic ulcers, like any other chronic wound, pose formidable clinical challenges, marked by the wounds’ inability to progress through the usual stages of healing and becoming trapped in the inflammatory phase. This prolonged inflammation stems from sustained tissue damage, which triggers a continuous influx of cells and intensifies the pro-inflammatory cytokine cascade [1,2]. An imbalance between proteases and their inhibitors further exacerbates the situation, leading to the breakdown of the extracellular matrix (ECM) and the degradation of crucial growth factors and their receptors. As a result, the proteolytic destruction of the ECM hampers the ulcer’s progression into the proliferative phase, fostering the recruitment of additional inflammatory cells and perpetuating the cycle of inflammation [3,4].

In diabetic ulcers, human neutrophil elastase (HNE) emerges as a significant contributor to the impaired healing process because of its elevated concentrations compared to acute wounds. This heightened activity is a result of the prolonged inflammatory state characteristic of chronic wounds. As inflammation persists, more neutrophils are recruited to the wound site, leading to an increased secretion of HNE. Conversely, in wounds undergoing normal healing, the inflammatory response is only temporary. The disproportionate presence and activity of HNE in diabetic ulcers have profound implications for the healing process. HNE’s enzymatic activity targets various components critical for wound repair, including the ECM proteins and growth factors, both endogenous (naturally produced by the body to facilitate healing) and supplemental (applied externally to aid in wound repair). This degradation hampers the wound healing process, impeding tissue regeneration [1,5,6,7]. In efforts to counteract the harmful effects of HNE in diabetic ulcers, researchers have explored diverse therapeutic avenues, among which is the use of the tetrapeptide alanine–alanine–proline–valine (AAPV) [8]. AAPV acts as an antagonist to HNE, presenting a promising strategy for regulating its function. The hydrophobic sequence in AAPV shares structural similarities with amino acid regions present in elastin, a key component of the ECM [9]. Leveraging these structural resemblances, AAPV binds competitively to the active sites of HNE, the sub-sites P-P1, effectively obstructing its enzymatic activity. By limiting HNE’s actions, AAPV contributes to preserving the ECM’s structural integrity and safeguarding crucial growth factors essential for the ulcer healing process. Moreover, AAPV’s mechanism of action offers a targeted approach, specifically addressing the aberrant protease activity observed in diabetic ulcers without interfering with physiological processes in healthy tissues [10,11].

Despite its efficacy, the widespread pharmaceutical application of peptides faces obstacles, including limited stability in physiological settings and susceptibility to environmental factors. However, AAPV’s overall hydrophilicity (despite the presence of hydrophobic domains) tends to reduce its bioavailability and cellular tissue permeability. The attachment of hydrophobic carriers/molecules can overcome these limitations [11,12]. Tryptophan (W) is an aromatic hydrophobic amino acid, which has been associated with the activation of antibacterial properties (disturbance of bacterial membrane), when incorporated into peptide sequences [13]. Considering that the abnormal response of HNE is aggravated by the presence of bacteria pathogens in the ulcer bed, the addition of antibacterial amino acids to therapeutic peptides may offer supplemental opportunities for promoting healing. According to Miranda et al., AAPV can be considered an antimicrobial peptide (AMP). However, its effectiveness is limited to concentrations over 2 mg/mL and its activity against bacteria relies on those increased amounts to isolate the cells and block nutrient exchanges or other biological factors essential to the bacteria survival [8]. Attending to this information, in this investigation, the incorporation of tryptophan at the N-terminal of the peptide, generating WAAPV, was postulated for improving the AAPV’s permeability and antibacterial capacity while maintaining its activity against HNE.

For effective topical delivery, an electrospun fibrous mat of polycaprolactone (PCL)/polyethylene glycol (PEG), loaded with the formulated peptide sequences, was developed and used as a carrier. PCL is an anti-adhesive polymer, mechanically resilient, and flexible, while PEG is highly versatile, cost-effective, and hygroscopic (it has also been labeled as a bacterial repellent); both have been identified as safe by the US Food and Drug Administration [14,15,16,17]. The electrospinning technique has stood out for its versatility in producing micro- and nanofibrous systems with a large surface area, controlled permeability, and intricate porous structure, resembling the skin’s ECM (which has also been obtained with the combination PCL/PEG) [1,2,18,19]. Additionally, the reduced pore size and high fibrous density of these systems condition bacterial infiltration. Through this structure, a topical release of AAPV and WAAPV peptides can be ensured for effective treatment of early- or advanced-stage diabetic ulcers. Furthermore, by demonstrating the inhibitory potential of WAAPV against HNE and bacteria, the first steps towards establishing this peptide as a candidate/model for new multi-action therapies in diabetic ulcers’ care can be taken.

## 2. Materials and Methods

### 2.1. Materials

The polymers PCL (Mw 80,000) and PEG (Mw 300 Da) were purchased from Sigma-Aldrich (Schnelldorf, Germany), and the reagents *N,N*-dimethylformamide (DMF) and chloroform (CHF) were obtained from PanReac AppliChem, ITW Reagents (Italy). Ethyl cyanoglyoxylate-2-oxime (Oxyma), 1,3-diisopropylcarbodiimide (DIC), dichloromethane (DCM), chloroacetic acid, acetic acid (AcOH), deuterium oxide (D_2_O), elastase from human leukocytes, trypsin inhibitor from soybean, and *N*-Methoxysuccinyl-Ala-Ala-Pro-Val-p-nitroanilide (N-MeO-Suc-Ala-Ala-Pro-Val-p-NA) were also purchased from Sigma-Aldrich. Tris-hydrochloride (Tris-HCl) was purchased from Roche (Basel, Switzerland). 2-Chlorotritylchloride resin and *N*-fluorenylmethyloxycarbonyl (Fmoc) amino acids were acquired from AAPPTec Peptide (Louisville, KY, USA). Acetonitrile (ACN), piperidine, 2,2,2-trifluoroethanol (TFE), methanol (MeOH), and trifluoroacetic acid (TFA) were acquired from Merck (Darmstadt, Germany). Also, triisopropylsilane (TIPS) and 2,4,6-trinitrobenzenesulfonic acid (TNBS used at 1% *v*/*v* in DMF) were obtained from Fluorochem (Glossop, UK) and diethyl ether from Fisher Scientific (Portsmouth, NH, USA). Sodium phosphate dibasic (Na_2_HPO_4_, Sigma-Aldrich), monosodium phosphate monohydrate (NaH_2_PO_4_, Merck), and sodium chloride (NaCl, Merck) were utilized in the formulation of a phosphate-buffered saline solution (PBS at 0.01 M concentration: 1.44 g/L Na_2_HPO_4_, 0.24 g/L KH_2_PO_4_, 0.20 g/L KCl, and 8.00 g/L NaCl, adjusted to a physiological pH of 7.4). The Gram-positive bacterium *Staphylococcus aureus* (American Type Culture Collection, ATCC 6538) and the Gram-negative bacterium *Escherichia coli* (ATCC 25922), both reference bacterial strains, were sourced from the ATCC (Manassas, VA, USA). Trypticase soy broth (TSB) and trypticase soy agar (TSA) for bacterial growth were procured from VWR (Alfragide, Portugal), while Mueller Hinton broth (MHB) was obtained from CondaLab (Madrid, Spain). All reagents and polymers were used as received from commercial suppliers, without further purification.

### 2.2. AAPV and WAAPV Synthesis and Characterization

Peptides were synthesized using solid-phase peptide synthesis with a 2-chlorotritylchloride resin pre-loaded with valine (functionalized at 0.53 mmol/g for AAPV and 0.918 mmol/g for WAAPV). The coupling steps involved Fmoc amino acids (5.0 equivalents relative to the resin functionalization), DIC (5.5 equivalents), and Oxyma (5.5 equivalents) in dry DMF (6 mL/g resin) with stirring at room temperature (RT) for 6 h. After each coupling, the Fmoc group was removed with 20% piperidine in DMF (6 mL/g resin), and the resin was washed with DMF (3 × 3 mL) and MeOH (3 × 3 mL). This washing cycle was repeated four times. Peptides were cleaved from the resin using a cleavage cocktail of AcOH/TFE/DCM in a 1/2/7 (*v*/*v*/*v*) ratio (10 mL/g resin). The side chain Boc protecting group used in WAAPV synthesis was removed using the cleavage cocktail TFA/TIPS/distilled water (dH_2_O), 95/2.5/2.5 (*v*/*v*/*v*) (10 mL/g resin). The peptides were precipitated from the reaction mixture with cold diethyl ether, centrifuged, and dried.

The peptides’ purity was checked by analytical High-Performance Liquid Chromatography (HPLC) using a Merck Licrospher RP-18 column (Darmstadt, Germany), a Jasco pump PU-980, and detector UV-975 and Shimadzu C-R6A chromatopac (Shimadzu Europa GmbH, Duisburg, Germany). An ACN/dH_2_O mixture at 1/1 (*v*/*v*), with 0.1% (*v*/*v*) TFA, was used as an eluent and detected at 214 nm. The peptides’ structure was confirmed with ^1^H, ^13^C, and two-dimensional Nuclear Magnetic Resonance Spectroscopy (NMR, Bruker Avance III 400, Fällanden, Switzerland), using 20 μL TFA per mL of DMSO-d_6_ as a solvent. All chemical shifts are given in ppm using tetramethylsilane as a reference and *J* values are given in Hz. AAPV NMR characterization has been reported in [8].

The secondary structure of the peptides was evaluated using a Jasco J-1500 spectropolarimeter, equipped with a temperature controller. The peptide concentration was set at 0.5 mM dissolved in dH_2_O. The baseline was recorded using the same conditions and subtracted to the peptide spectrum. The spectra were recorded between 180 and 260 nm at a scan speed of 100 nm/min and bandwidth of 1 nm. The path-length cell was set to 1 mm. The final spectra were obtained by the average of three scans for each sample and processed through smoothing using the Savitzky–Golay method.

^1^H NMR (DMSO-d_6_ + TFA, 400 MHz) δH: 0.84 (6H, d, 2×γ-CH_3_ Val), 1.21 (3H, dd, *J* = 7.2 Hz e 10.4 Hz, CH_3_ Ala 2), 1.21 (3H, dd, *J* = 7.2 Hz e 10.4 Hz, CH_3_ Ala 1), 1.76–1.93 (3H, m, γ-CH_2_ Pro + β-CH_2_ Pro), 1.93–2.06 (2H, m, γ-CH_2_ Pro + β-CH Val), 3.02 (1H, dd, *J* = 8.8 Hz e 15.2 Hz, β-CH_2_ Trp), 3.23 (1H, dd, *J* = 5.2 Hz e 14.8 Hz, β-CH_2_ Trp), 3.49–3.63 (2H, m, δ-CH_2_ Pro), 4.02 (1H, q, α-CH Trp), 4.08 (1H, dd, *J* = 5.6 Hz e 8.4 Hz, α-CH Val), 4.37 (1H, q, *J* = 7.2 Hz, α-CH Ala 1), 4.44 (1H, dd, *J* = 3.2 Hz e 8.4 Hz, α-CH Pro), 4.50 (1H, q, *J* = 6.8 Hz, α-CH Ala 2), 6.98 (1H, dt, *J* = 1.2 Hz e 7.6 Hz, H-5 Trp), 7.07 (1H, dt, *J* = 1.2 Hz e 7.6 Hz, H-6 Trp), 7.20 (1H, d, *J* = 2.4 Hz, H-2 Trp), 7.35 (1H, d, *J* = 8.0 Hz, H-7 Trp), 7.67 (1H, d, *J* = 8.0 Hz, H-4 Trp), 7.89 (1H, d, *J* = 8.8 Hz, NH Val), 7.98 (2H, br s, ^+^NH_3_ Trp), 8.17 (1H, d, *J* = 7.2 Hz, NH Ala 2), 8.72 (1H, d, *J* = 7.6 Hz, NH Ala 1), 10.95 (1H, d, *J* = 2.0 Hz, NH Trp).

^13^C NMR (DMSO-d_6_ + TFA, 100.6 MHz) δC: 17.2 (CH_3_ Ala 2), 18.5 (CH_3_ Val), 18.1 (CH_3_ Ala 1), 19.3 (CH_3_ Val), 24.8 (γ-CH_2_ Pro), 27.7 (β-CH_2_ Trp), 29.0 (β-CH_2_ Pro), 30.3 (β-CH Val), 46.6 (α-CH Ala 1), 47.0 (δ-CH_2_ Pro), 48.5 (α-CH Ala 2), 53.8 (α-CH Trp), 57.5 (α-CH Val), 59.3 (α-CH Pro), 107.1 (C-3 Trp), 111.8 (C-7 Trp), 118.7 (C-5 Trp), 118.8 (C-4 Trp), 121.5 (C-6 Trp), 125.4 (C-2 Trp), 127.4 (C-3a Trp), 136.7 (C-7a Trp), 168.4 (C=O Trp),170.9 (C=O Ala 2), 171.5 (C=O Ala 1), 172.0 (C=O Pro), 173.3 (C=O Val) ppm.

### 2.3. Inhibition of Elastase and Bacteria Activities

The inhibition of HNE activity caused by both AAPV and WAAPV peptides was evaluated by following a protocol described in research conducted by Melzig et al. [20]. Initially, 125 µL of a substrate solution consisting of N-MeO-Suc-Ala-Ala-Pro-Val-p-NA dissolved in a Tris-HCl buffer was mixed with 405 µL of a 0.1 M Tris-HCl buffer solution. Afterwards, 50 µL of testing solutions composed of AAPV and WAAPV dissolved in a Tris-HCl buffer at concentrations from 0.10 to 200.00 µg/mL was also disposed into the mixture, followed by an addition of 20 µL of an enzyme solution (HNE diluted in Tris-HCl buffer at 45 mU). The mixtures were then vortexed for 1 min and incubated for 1 h at 37 °C. The concentration of the HNE solution was based on the literature, according to which, the concentration of such an enzyme in diabetic ulcers varies between 36 and 54 mU/mL [21].

Finally, 500 µL of an inhibitor solution (trypsin soybean inhibitor, also diluted in Tris-HCl buffer) was added so that the reaction would be stopped. Absorbances were then read at 405 nm with the use of an EZ Read 2000 Microplate Reader. Control samples without any of the testing peptides were also tested and experiments were conducted in three replicates per sample, each replicate with three absorbance readings (mean averaging nine measurements). Results were presented as maximum inhibitory concentration (IC_M_), corresponding to the concentration causing the highest inhibition of HNE activity.

Minimum inhibitory concentration (MIC) of both peptides and PEG was determined, against *S. aureus* and *E. coli* by the broth microdilution method [22], based on a publication by the Clinical and Laboratory Standards Institute (CLSI) and European Committee on Antimicrobial Susceptibility Testing (EUCAST) [18]. For this test, AAPV and WAAPV were dissolved in dH_2_O at a concentration of 4.096 mg/mL, whereas PEG was also diluted in dH_2_O at 10.240 mg/mL, working as stock solutions. Afterwards, 100 µL from each stock solution was added to the first column of a 96-well plate and serial dilutions (1/2 *v*/*v*) were carried out in MHB, until a final volume of 50 µL was achieved in each well. Then, a bacterium suspension at 1 × 10^6^ CFUs/mL was added to the wells. Controls consisting of MHB and bacteria suspensions free from any of the active agents were produced, representing the negative and positive control samples, respectively. Absorbance readings were conducted with an EZ Read 2000 Microplate Reader at 600 nm before (0 h) and 24 h after incubation at 37 °C at 120 rpm. MIC was then perceived by the differences between the several absorbance readings. Minimum bactericidal concentrations (MBCs) were established by the culture of the same bacterium suspensions at MIC and their vicinities (dilutions before and after MIC value). Aliquots were collected and diluted in PBS (10^−1^–10^−4^). Then, the aliquots were plated in TSA and incubated at 37 °C during 24 h, after which the grown colonies were observed and counted.

### 2.4. Electrospun Fiber Production and Modification

PCL was solubilized at 14% *w*/*v* in CHF/DMF (9/1 *v*/*v*) under constant stirring at 150 rpm and 50 °C for 4 h. The PCL/PEG solution was prepared from a PCL solution (14% *w*/*v*; prepared as described earlier) to which PEG was added at the determined MBC concentration against *S. aureus* and *E. coli* bacteria. The solution containing both polymers was allowed to dissolve for 1 h at approximately 30 °C with constant stirring at 150 rpm. The AAPV peptide was added to the polymeric solutions at IC_M_. WAAPV was added at the same concentration as AAPV (even though it was not the IC_M_ of WAAPV) for comparison purposes between peptides. The peptides were allowed to dissolve on both PCL and PCL/PEG solutions overnight at RT. The viscosities of both PCL and PCL/PEG, with and without the peptides, were measured using a Brookfield DV-II + Pro viscometer (Hadamar-Steinbach, Hesse, Germany) with spindle 21, operating at 15 rpm at approximately 20 °C for 10 min, with data being recorded every 30 s. Conductivities were measured with a Thermo Scientific Benchtop Meter (Orion Versa Star Pro, Waltham, MA, USA). Following optimization, a consistent electrospinning voltage of 12 kV was applied to a steel capillary needle (single nozzle) with an inner diameter of 18 Gauge (G). The solution feeding rate was set to 0.7 mL/h, and an aluminum collection sheet was placed 17 cm from the needle tip for fiber collection. The electrospinning process was conducted under controlled conditions, with the temperature being maintained at 20–22 °C and relative humidity (RH) at 60–65%. Fibers were stored in a desiccator for at least 24 h prior to any usage, for the complete elimination of solvent molecules still present. Peptide loading was determined by measuring the volume of the polymeric solutions required to produce a given amount (mass and area) of electrospun fibers.

The nomenclature of the samples was defined based on the composition, where the symbol “/” was used to indicate combinations of polymers and “+” was used to indicate the addition of the peptide: PCL + AAPV, PCL + WAAPV, PCL/PEG + AAPV, PCL/PEG + WAAPV.

### 2.5. Physical, Chemical, and Thermal Characterization

Each mat typology was evaluated using the following methods: scanning electron microscopy (SEM), Fourier-transform infrared spectroscopy coupled with attenuated total reflection (ATR-FTIR), thermogravimetry (TGA), wettability (goniometer), swelling capacity (mass variations), and degradation profile (mass variations).

Micrographs of the electrospun fibers were captured using a field emission gun scanning electron microscope (FEG-SEM, NOVA 200 Nano SEM, FEI Company, Hillsboro, OR, USA) with an accelerating voltage of 10 kV. Before imaging, the fibrous mats were coated with a thin layer (10 nm) of Au-Pd (80–20 wt.%) using a 208 HR high-resolution sputter coater (Cressington Company, Watford, UK) paired with an MTM-20 Cressington High Resolution Thickness Controller. To determine the average fiber diameters, 50 measurements were taken on three micrographs of each sample, and the diameter distribution was modeled with a log-normal function. Images at a 10,000× magnification were obtained and analyzed using ImageJ software (version 1.51j8, National Institute of Health, Bethesda, MD, USA).

The chemical composition of the fibers was analyzed using Fourier-transform infrared spectroscopy with attenuated total reflection (ATR-FTIR) with an IRAffinity-1S spectrometer (Shimadzu, Kyoto, Japan), equipped with an HATR 10 accessory and a diamond crystal. Spectra were recorded over a range of 400 to 4000 cm^−1^, with 200 scans at a resolution of 2 cm^−1^.

Thermal responses of the fibers were assessed by a thermogravimetric analysis (TGA). Weight loss was monitored as the temperature increased from 25 to 600 °C at a heating rate of 10 °C/min under a dynamic nitrogen atmosphere with a flow rate of 200 mL/min, using an STA 7200 instrument (Hitachi^®^, Fukuoka, Japan) and aluminum pans. Results were plotted as the percentage of weight loss vs. temperature. Water contact angles were determined using an OCA 200 goniometer, Data Physics (Filderstadt, Germany), following an adaptation of the ASTM-D7334-08 standard [23]. Droplets of 5.0 µL of dH_2_O, ejected at a rate of 2.0 µL/s, were used to measure the fibers’ wettability via the sessile drop method. Six measurements were conducted per type of sample. Angles were recorded immediately after drop contact with the surface (≈10 s after contact).

The fibers’ capacity for air exchange (permeability) was examined according to standard ISO 9237 [24]. Air pressure of 200 Pa was applied on three samples of each typology in three randomly selected points of 1.33 cm^2^ (1.30 cm diameter), and the permeability was measured. For determining the porosity (P%) of the samples [25], measurements of the amount of ethanol absorbed after 1 h of immersion in static conditions and RT (Equation (1)) were used:

(1) $$P(\%)=\frac{W_{w}-W_{D}}{\rho_{Ethanol}V_{Sample}}\times 100,$$

where W_W_ and W_D_ are the weight of the wet and dry samples, respectively; ρ_ethanol_ is the density of the ethanol at RT (0.789 g/cm^3^); and V_sample_ is the volume of the wet sample, determined by measuring the 6 mm diameter samples’ thickness with a pachymeter. The experiment was performed in triplicate.

The degree of swelling (DS) of the fibrous mats was determined by measuring the weight of the samples (6 mm diameter) before and after 4, 6, 8, and 24 h of incubation in PBS at 37 °C (static conditions). Prior to weighing, excess PBS was removed from the surface using Kimwipes (Kimtech). The degree of swelling was calculated using the following equation (Equation (2)) and was expressed as a percentage:

(2) $$DS(\%)=\frac{m_{w}-m_{d}}{m_{w}}\times 100,$$

where m_w_ (mg) is the weight of the wet sample after the incubation period and m_d_ (mg) is the weight of the dry sample.

The degradation profile of the electrospun fibers (6 mm diameter) was monitored in PBS (500 µL) at 37 °C for a maximum period of 7 days. The test was conducted in static conditions. Initially, the samples were placed in 500 µL of PBS for 24 h in a refrigerator for pre-hydration (4 °C), serving as the starting point (time: 0 h). After this period, the medium was removed and the samples were weighed, and fresh PBS was added. At the end of 1, 2, 3, 4, 5, 6, and 7 days of incubation, excess PBS was removed from the surface of the samples using Kimwipes, and the samples were weighed. Degradation, measured in mass loss [26], was calculated using the following equation (Equation (3)):

(3) $$massloss(\%)=\frac{m_{ti}-m_{tf}}{m_{ti}}\times 100$$

where m_ti_ (mg) is the weight of the sample on day 0 (after 24 h hydration) and m_tf_ (mg) is the weight of the sample after each incubation period.

### 2.6. Peptide Release Kinetics

A WAAPV calibration curve in PBS at concentrations between 0.500 and 62.500 µg/mL was elaborated using a Spectrometer Sarspec FLEX (Vila Nova de Gaia, Portugal) and LightScan 2.0 software. WAAPV-loaded mats were immersed in PBS for 1, 2, 4, 6, and 24 h of incubation at 37 °C and 120 rpm, and aliquots of 150 µL were collected at each time point. Fluorescence was then read with an LED lamp of 275 nm, in the range 200–900 nm. Fluorescence of WAAPV-unloaded mats was also collected to subtract PEG and/or PCL influence on the release profiles. Results were reported as intensity counts vs. wavenumber, based on the WAAPV calibration curve.

Due to the absence of any fluorescence from AAPV, along with an overlapping of maximum absorbances between the fiber compounds when analyzed by UV–visible spectroscopy, AAPV release kinetics tracking could not be conducted.

### 2.7. HNE Inhibition

In order to evaluate the HNE inhibitory capacities from all the produced mats, AAPV- and WAAPV-loaded fibers were exposed to PBS for 1, 2, 4, 6, and 24 h at 37 °C and 120 rpm. Aliquots of 150 µL were collected after each time point, constituting the testing solutions. Afterwards, HNE inhibition tests were carried out as described in Section 2.3. Absorbances were then read at 405 nm (EZ Read 2000 Microplate Reader) and data were presented as IC_M_. All experiments were performed in triplicate in which three absorbance readings were performed for each replicate (mean averaging nine measurements).

### 2.8. Antibacterial Activity

The time-kill kinetics test, adapted from the standard ASTM E2149-20 [27], was employed to determine the efficacy of both PEG and AAPV or WAAPV peptides loaded onto the electrospun mats in eliminating bacteria. The antibacterial profile of the mats was evaluated against suspensions of *S. aureus* and *E. coli* (representatives of Gram-positive and Gram-negative bacteria, respectively) prepared in TSB at 1 × 10^5^ CFUs/mL. Samples of 1 × 1 cm^2^ mats were immersed in 500 µL of a bacterial suspension and incubated at 37 °C and 120 rpm for 24 h. After this period, 20 µL aliquots of the bacterial suspension were withdrawn and serially diluted in PBS (10^−1^ to 10^−5^), plated on TSA-filled Petri dishes (90 mm diameter), and incubated at 37 °C for another 24 h. The grown colonies were counted (averaging data from 3 replicates times 3 repetitions), and the results were expressed as the percentage of bacterial reduction.

## 3. Results and Discussion

### 3.1. Peptide Synthesis and Characterization

**Figure 1:**
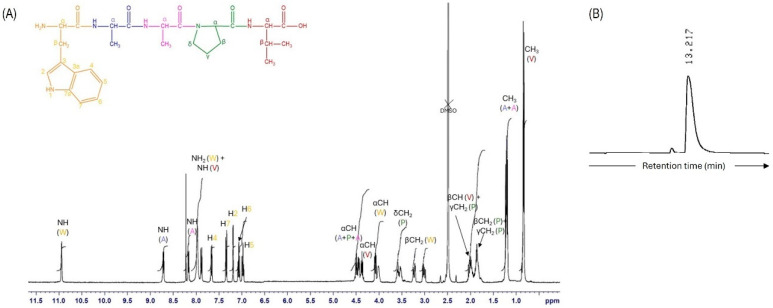
(**A**) ^1^H NMR spectra of WAAPV in DMSO-d_6_ (color coded according to the chemical structure above). (**B**) WAAPV HPLC partial chromatogram at 214 nm.

AAPV was obtained as a white powder in 94% yield (0.131 g) and 98% purity. Data on the synthesis and characterization (NMR and HPLC) of this tetrapeptide have been detailed in [8]. On its turn, WAAPV was also obtained as a white powder solid in 68% yield (0.365 g) and 96% purity, estimated by HPLC. All the couplings proceeded to completion, as confirmed by the TNBS test; the lower yield obtained with WAAPV was due to difficulties in recovering the peptide from the cleavage reaction mixture. The structural characterization was accomplished by NMR (Figure 1A), with all expected signals being observed. The presence of tryptophan was easily verified by the presence of the characteristic aromatic protons. WAAPV purity was determined by HPLC with detection at 214 nm, a retention time of 13.2 min, and a flow rate of 1 mL/min (Figure 1B). Both peptides were considered pure (≥95%). Also, circular dichroism spectra were obtained for AAPV and WAAPV. As expected for such short sequences, the results indicate a preference for a random coil with the characteristic negative and positive bands, at about 195 and 220 nm, respectively, for both peptides. This result demonstrates that the secondary structure of AAPV was not significantly altered by the inclusion of tryptophan, meaning that similar activities are expected from the two peptides.

### 3.2. HNE Inhibition by AAPV and WAAPV

Several solutions of AAPV and WAAPV were prepared at concentrations ranging from 0.10 to 200.00 μg/mL, to evaluate the HNE inhibitory capacities of both peptides (Table 1). According to Edwards et al., the interactions between AAPV and HNE result from similarities between the hydrophobic sequences of the peptide and amino acid regions of elastin, which are susceptible to HNE activity. Consequently, the peptide binds to HNE subsites and leads to a competitive HNE inhibition [21]. To the authors’ knowledge, there are still no reports on the interactions between WAAPV and HNE. However, it is likely that such interactions may occur similarly to those between AAPV and HNE. In an investigation conducted by Marinaccio et al., brunsvicamide C, whose main compound is the *N*-methyl-*N*-formylkynurenine derived from tryptophan, was found to inhibit HNE [28]. In general, WAAPV presented slightly higher HNE inhibitory effects in comparison with AAPV, likely caused by the simultaneous inhibitory capacities of AAPV and the presence of tryptophan (Table 1). Another plausible explanation relies on the hydrophobic nature of tryptophan that resulted in higher bioavailability and cellular tissue permeability of WAAPV, thus enhancing its enzymatic activity [13].

AAPV presented its maximum inhibitory capacity at a concentration of 50 μg/mL, which is in accordance with previous research conducted by our team [8]. According to Namjoshi et al., the ability of HNE to digest substrates decreases with increasing inhibitor concentrations [11]. This same occurrence may result from a competition between the AAPV molecules (superior amount at concentrations of 200 and 100 μg/mL), compromising the effective binding to HNE sites, thus reaching an equilibrium between peptide molecules and the available enzymatic sites when AAPV is present at 50 μg/mL [8]. Such a theory is also supported by the fact that the inhibitory capacities of the peptide were inferior at concentrations lower than 50 μg/mL, since there were insufficient peptide molecules to bind to all enzymatic sites.

On its turn, WAAPV also attained a maximum inhibitory capacity at a concentration of 50 μg/mL. However, the peptide presented another maximum value when loaded at a concentration of 1.56 μg/mL. Such difference in outcomes between the peptides may be caused by the presence of tryptophan, whose mechanism of action is not yet fully understood. Interestingly, in the research of Marinaccio et al., the tested molecule of brunsvicamide C with an amino acid derived from tryptophan achieved IC_50_ values as low as 3.12 μM [28]. Still, the WAAPV loading amount was established at 50 μg/mL since the inhibitory capacities achieved at the two concentrations were not considered significantly different. Also, the loading on the mats at equal concentrations would enable a direct comparison between the enzymatic activities of AAPV and WAAPV.

### 3.3. Minimum Inhibitory and Bactericidal Concentrations

MIC and MBC were determined for the two peptides, AAPV and WAAPV, and for the polymer PEG against *S. aureus* (Gram-positive bacterium) and *E. coli* (Gram-negative bacterium). This is the first report on WAAPV antibacterial potential, and the first examination of AAPV activity against the *E. coli* bacterium. For both peptides, MIC and MBC were established at 2.048 mg/mL against *S. aureus* and 4.096 mg/mL against *E. coli*, revealing a cost-ineffective antibacterial activity (considering the elevated cost of peptide production). Even though the antibacterial mechanisms of action of these peptides have never been established, previous work by our team on small-sized peptides postulated that they may be capable of permeating the cells through the bacterium membrane, engaging in detrimental interactions with intracellular components, or to encircle and isolate the bacterial cells (considering the large concentrations determined for MBC), blocking specific activities vital for bacterial survival [8]. This last mechanism is also proposed to explain PEG’s activity. This polymer reported an MIC of 64 mg/mL and MBC of 128 mg/mL against *S. aureus* and MIC and MBC of 256 mg/mL against the *E. coli* bacterium. PEG is often labeled as biologically inert and non-antimicrobial [29]; yet, low-molecular-weight PEG has been reported to exhibit a repellent function, protecting against the penetration of bacteria [30].

In both peptide and polymer testing, *E. coli* was the bacterium less susceptible to the action of the bioactive agents. Differences in cell wall structures and compositions between Gram-positive and Gram-negative bacteria are behind these observations. Gram-positive bacteria possess a single thick layer of peptidoglycans, forming a rigid three-dimensional structure with linear polysaccharide chains crosslinked with peptides. In contrast, Gram-negative bacteria feature a structurally and chemically complex cell wall comprising a thin layer of peptidoglycans adjacent to the cytoplasmic membrane and an outer lipopolysaccharide membrane. Active agent penetration occurs through porins in the external membrane, restricting the size and structural conformation of molecules that can pass through [31,32]. Considering the large MBCs, peptide loading onto the electrospun fibers was selected based on data from Section 3.2. On the other hand, PEG concentration for incorporation in the PCL/PEG fibers was established at 256 mg/mL (MBC that works against both Gram-positive and Gram-negative bacteria; PCL/PEG *w*/*w* ratio of 1.00/2.56).

### 3.4. Physical, Chemical, and Thermal Characterization of Electrospun Mats

#### 3.4.1. Morphology

**Figure 2:**
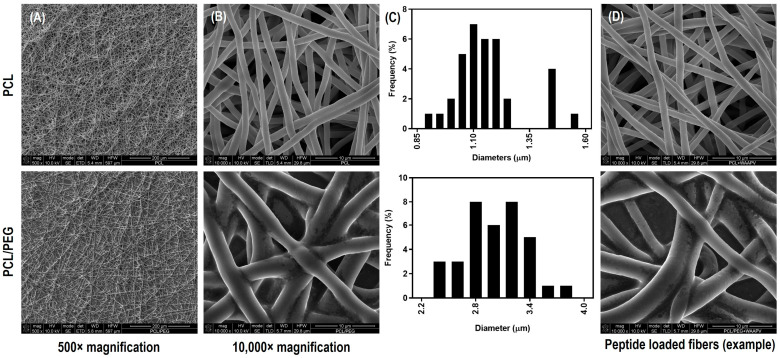
FEG-SEM micrographs of PCL and PCL/PEG electrospun fibers (**A**) at 500× and (**B**) 10,000× magnification. (**C**) Fiber diameter distribution histograms of PCL and PCL/PEG fibers. (**D**) FEG-SEM micrographs representative of PCL and PCL/PEG fibers modified with peptides, in this case the WAAPV, with no visible alterations in morphology.

FEG-SEM micrographs of PCL and PCL/PEG electrospun fibers (using solutions with ≈1.11 µS/cm for PCL and ≈3.98 µS/cm for PCL/PEG in conductivity), with and without the incorporation of the peptides, are shown in Figure 2. Continuous, homogeneous, and defect-free fibers were attained with all testing conditions, independent of the presence and absence of the peptides. PCL fibers reported an average diameter of 1.23 ± 0.21 µm, which is consistent with work previously performed by our team [33]. On its turn, PCL/PEG fibers were found to be larger, as clearly depicted in Figure 2C,D, with diameters averaging 2.91 ± 0.41 µm. This increase as two- to three-fold in fiber diameter can be attributed to the freedom and flexibility of the polymeric chains introduced by PEG (a plasticizing agent), which also reduced the viscosity of the polymeric solution (from 1.13 ± 0.11 Pa.s for PCL to 0.70 ± 0.07 Pa.s for PCL/PEG) [34]. Also, because of PEG’s hydrophilic nature, it migrates towards the outside regions of the fibers, promoting an outward expansion [35]. As expected, the small amount of peptide introduced in the fibrous system (concentration defined based on HNE inhibitory studies and calculated based on volume ejected during electrospinning and sample mass in 51.1 ± 2.3 µg/cm^2^ and 46.0 ± 5.8 µg/cm^2^ for AAPV and 48.5 ± 1.2 µg/cm^2^ and 51.3 ± 3.6 µg/cm^2^ for WAAPV, respectively, for PCL and PCL/PEG) did not influence the fibers’ morphology nor dimension.

#### 3.4.2. Chemical Composition

**Figure 3:**
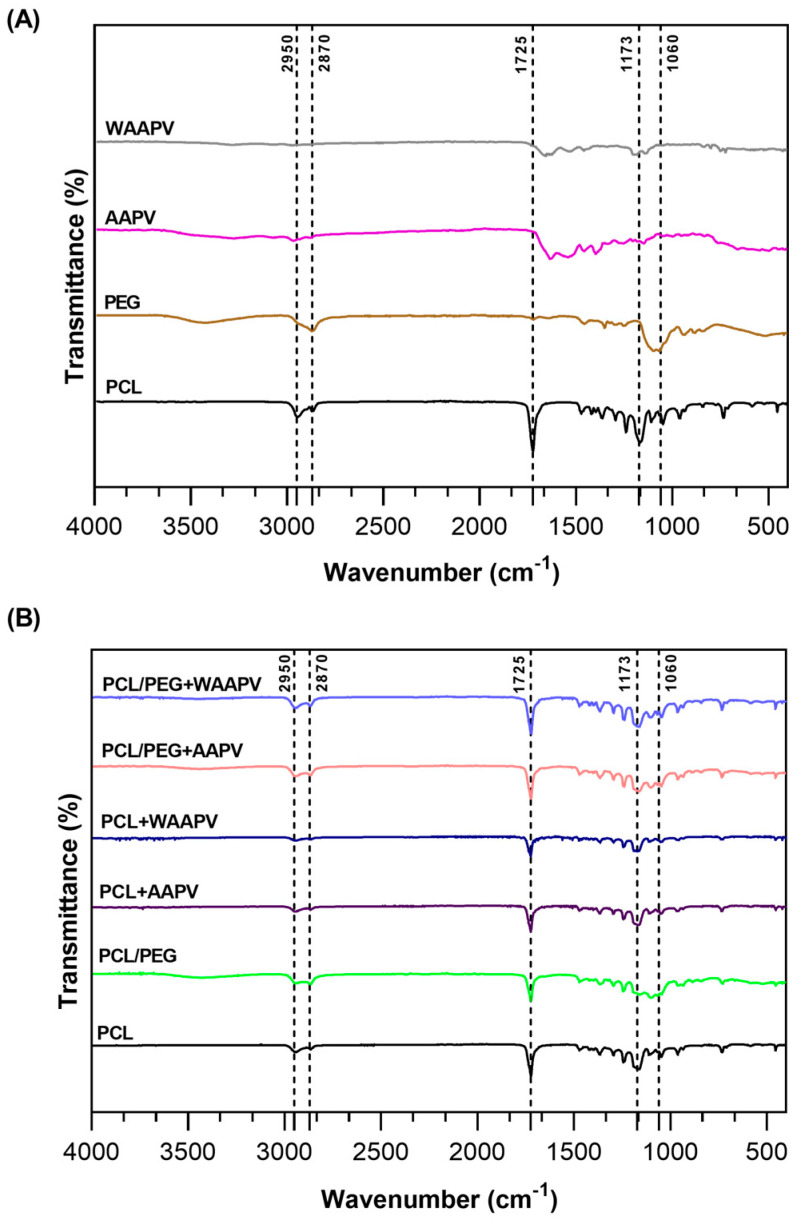
ATR-FTIR spectra of (**A**) the peptides and polymer powders, and (**B**) the PCL and PCL/PEG fibers unloaded and loaded with the two peptides.

The spectral analysis conducted via ATR-FTIR encompassed both control samples (powder forms, before electrospinning processing) and the fibrous mats, as depicted in Figure 3. For individual PCL polymer identification and for samples where PCL was combined with PEG or the peptides, characteristic peaks confirmed its presence. These included a peak at 1725 cm^−1^ attributable to C=O bond vibrations (carbonyl group), along with peaks at 2950 and 2870 cm^−1^ indicative of C-H bonds, and a peak at 1173 cm^−1^ associated with C-O-C bond vibrations [36,37]. The addition of PEG in the mats was substantiated by a peak at 2870 cm^−1^ corresponding to C-H bonds and another at approximately 1060 cm^−1^ characteristic of C-O-H bonds [35]. PEG contributed to a slight attenuation of the PCL peaks at 1725 cm^−1^ and 1173 cm^−1^, in terms of area dimension, probably a result of the organization of the polymer chains; however, it was non-significant. Because of the low concentration of the loaded AAPV and WAAPV compared to other polymers in the fibers, their detection was easily overshadowed by PCL and PEG. Therefore, while ATR-FTIR provided valuable insights into the composition of the systems, limitations regarding the detection sensitivity of the peptides were acknowledged.

#### 3.4.3. Thermal Response

**Figure 4:**
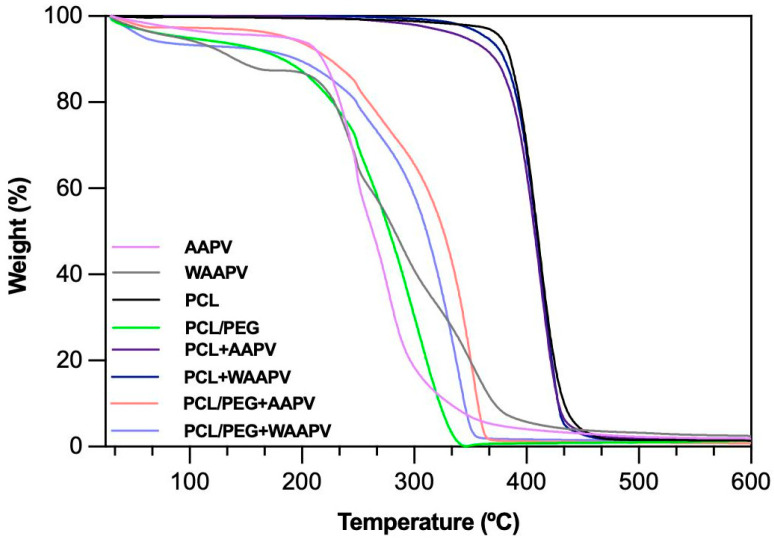
TGA spectra of the free peptides and the PCL and PCL/PEG fibers, unloaded and loaded with AAPV and WAAPV, obtained under a nitrogen atmosphere of 200 mL/min and heating rate of 10 °C/min.

The thermal degradation behaviors of the peptides and electrospun fibers were characterized using TGA (Figure 4). Prior to the analysis, all samples underwent a drying process at 50 °C for 1 h to mitigate the effects of surrounding environment humidity. PCL’s thermal stability was constant up until ≈370 °C, the typical behavior for this polymer [36]. Beyond 370 °C and up until ≈435 °C, thermal degradation primarily affected the polymeric structure, commencing with side chain breakdown and progressing to main chain cleavage, with minimal weight loss observed up to 600 °C [38]. The influence of the peptides loading onto the PCL fibers was very small. However, there was a clear distinction between loading AAPV, a hydrophilic peptide, and WAAPV, a hydrophobic peptide. Because WAAPV can establish hydrophobic interactions with the hydrophobic polymer PCL and thus reaching the inner regions of the fibers and being more protected from external aggressions, thermal degradation started at ≈365 °C and reported polymer losses of the same rate as PCL (<4%) before the main step of degradation took place, therefore confirming the stability of the bond between the two elements. Yet, loading AAPV introduced alterations in the conformation of PCL, with the peptide being more exposed to the exterior due to its affinity with water, and leading thermal degradation to start earlier, at ≈ 335 °C, and material losses reached almost 8% compared to bare PCL (before the main degradation step was initiated). This is particularly important since the main degradation step for the peptides was around 200 °C with WAAPV being more sensitive to temperature variations than AAPV.

PCL/PEG formulations exhibited an initial degradation at around 50 °C, with ≈10% mass loss attributed to water evaporation, likely absorbed from the atmosphere after sample drying in response to PEG’s high water affinity [33,39]. Subsequently, thermal stability was maintained until roughly 190 °C, followed by progressive mass loss up to 380 °C. Because the chemical interactions with PCL are mostly in the form of ester bonds, enhancing chain flexibility and water affinity in the presence of PEG, thermal degradation was mostly influenced by PEG rather than PCL [40]. Notably, the presence of peptides influenced the thermal stability of the PEG-containing fibers (main degradation step starting at around 200 °C instead of the 190 °C reported for PEG), with AAPV promoting the highest thermal resistance (as confirmed by its thermogram). These results suggest the formation of stronger interactions between PEG and the peptides than with the PCL, and the shared hydrophilicity between PEG and AAPV to contribute more significantly to the thermal response of the electrospun fibers.

Generally, all tested polymer/peptide combinations demonstrated thermal resistance exceeding body temperature (>37 °C), ensuring potential stability when in contact with diabetic ulcers.

#### 3.4.4. Wettability

Static contact angle measurements are a conventional method to determine the wettability of surfaces. This method involves assessing the angle formed between the solid surface, the liquid droplet, and the vapor phase at the three-phase contact point. Based on these measurements, surfaces are classified into three categories: >150° is superhydrophobic, indicating an extreme resistance to wetting; from 90° to 150° is termed hydrophobic, suggesting a moderate resistance to wetting; and below 90° is categorized as hydrophilic, implying a propensity to be readily wet [41]. Electrospun fibers unloaded and loaded with the two peptides were examined for their affinity towards water. Data from Table 2 reported a hydrophobic behavior for PCL fibers, which was slightly reduced with the addition of the peptides, and a hydrophilic behavior for PEG. As a naturally hydrophilic polymer [35,40], PEG combined with PCL induced a structural rearrangement of the polymeric chains whereby the hydrophobic polymer migrated inward, while the hydrophilic component gravitated towards the outside of the fibers, in the direction of a water-containing environment [35]. Even though AAPV possesses hydrophobic domains, its overall structure is classified as hydrophilic [11,12]. On the other hand, tryptophan (W) is an aromatic hydrophobic amino acid, whose incorporation into peptide sequences increases their hydrophobic profile (WAAPV) [13]. The results in Table 2 expose this exact behavior: superior influence of the AAPV peptide, potentially being more exposed to the outside and thus raising the surface wettability, and a less impactful influence introduced by WAAPV, whose overall sequence is deemed hydrophobic just like the polymer PCL. Data confirm the successful incorporation of the peptides within the fibrous structure. Interestingly, the addition of AAPV and WAAPV to PCL/PEG generated a highly wettable surface, whose contact angle was undetectable as the water droplets were instantly absorbed by the surfaces. These results support our previous premise (TGA analysis) of the preferential interaction of the peptides with PEG, as this polymer has the biggest impact on the hydrophilicity of the electrospun fibers, potentially altering the polymer chains’ arrangement and offering a larger shielding to the PCL component and other hydrophobic domains.

#### 3.4.5. Air Permeability and Porosity

For effective diabetic ulcer healing, ensuring the proper oxygenation of the injured site and the surrounding blood supply is crucial. Thus, when designing a new dressing system, it is essential to consider the porosity of the structure for allowing air exchange [42]. Data from Table 3 indicated that most samples followed the trend of increased porosity that leads to enhanced air permeability, ensuring adequate oxygen flow. Microfibrous mats containing PEG were found to be more breathable. Despite the smaller fiber diameter in PCL fibers compared to PCL/PEG, PCL had a more densely packed fibrous structure, thus hindering air supply. Interestingly, the hydrophobic and hydrophilic nature of both polymers and peptides appeared to again influence the dressings’ properties. The association between both hydrophobic elements led to a reduction in porosity, turning the fibrous system more compact and less permeable, whereas fiber expansion and, consequently, larger porosity were evident with the combination of hydrophilic elements. In general, electrospun mats reported a very similar air permeability and porosity, potentially effective for inducing tissue regeneration in diabetic ulcers.

#### 3.4.6. Degree of Swelling

In diabetic ulcer care, guaranteeing the absorption of exudates and maintaining local moisture balance is essential for successful healing [43]. Here, the moisture retention capacity, termed DS, of the fibrous mats was assessed over a 24 h incubation period using physiological-mimicking media, the PBS (Table 4). Samples were weighed at various intervals (4, 6, 8, and 24 h) to pinpoint hydration equilibrium. Initially, all samples displayed high PBS absorption (>28%), with the lowest DS being reported for PCL and PCL + WAAPV, the most hydrophobic samples from the group (Table 2). This hydrophobic profile restricts interactions with water molecules due to electrostatic repulsion. On its turn, PCL/PEG fibrous mats, unloaded and loaded with the peptides, displayed great affinity towards PBS with DS at 4 h being >87%, hence corroborating the wettability observations and highlighting PEG’s hydrophilic nature influence over PCL’s hydrophobicity [39]. Additionally, AAPV superior water affinity compared to WAAPV was verified. Between the 8 h and the 24 h mark, minor mass fluctuations were detected on all samples, suggesting a constancy and, thus, absorption equilibrium. The tendency was for the mass of the samples to reduce from the 4 h to the 8 h testing period, suggesting binding and subsequent release of PBS salts preceded or followed by bonding with water, until optimal balance and bond stability were reached [44].

#### 3.4.7. Degradation Profile

The unloaded and peptide-loaded fibrous mats’ stability in PBS was monitored for up to 7 days of incubation at 37 °C. Degradation profiles were evaluated by tracking mass changes over time, as detailed in Table 5.

After exposure to PBS, PCL fibers experienced a continuous mass increment attributed to interactions between the polymer and PBS salts [45]. Conversely, PCL/PEG fibers, despite displaying higher wettability (Table 2) and, consequently, DS capacity (Table 4), demonstrated relatively modest mass losses, ranging between 10 and 20%, with the maximum recorded at 20.43 ± 0.28% on day 7. The compatibility of PEG with water and its solubility [39] contributed to the observed degradation-induced mass loss compared to PCL. However, as PEG possesses inherent bio-stability [46], losses remained below ≈ 20%. These findings suggest that degradation outweighs the interactions between PCL and PBS in PCL/PEG fibers.

In the presence of the peptides, their inherent hydrophilic and hydrophobic characters were once again significant on their influence over the polymers’ affinity towards the surrounding medium. It was evident that when combined with PCL, WAAPV contributed the most to the repellency of water molecules (no mass loss), while PCL + AAPV fibers experienced a small initial loss in mass, potentially associated with the release of the peptide. In the presence of PEG, the affinity of AAPV to water was again highlighted, this being the sample that experienced the largest loss in mass after 7 days of incubation. This demonstrates that even though the interaction between PEG and AAPV is strong, overtime, the surroundings’ influence becomes detrimental for preserving that stability.

### 3.5. Peptide Release Kinetics from Electrospun Fibers

**Figure 5:**
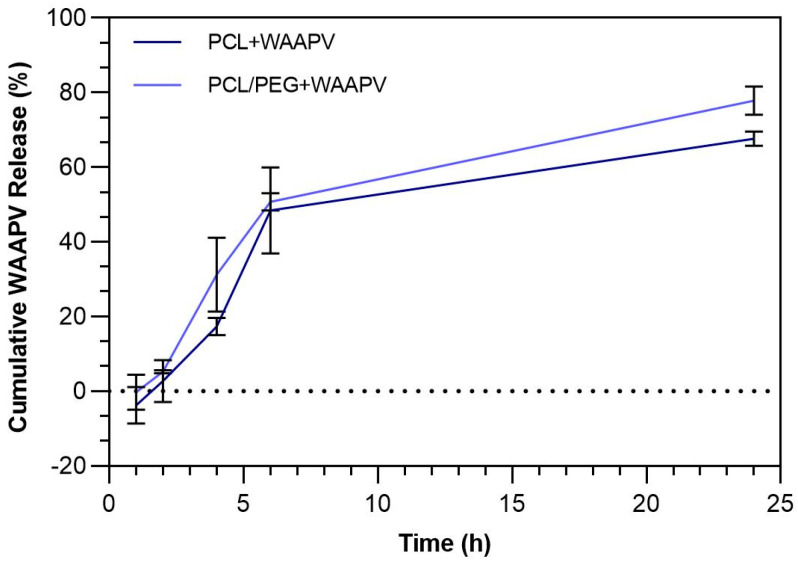
The release profile of WAAPV incorporated in the PCL and PCL/PEG fibers after 1, 2, 4, 6, and 24 h of incubation at 37 °C and 120 rpm in PBS.

The release of WAAPV, loaded at a concentration of 50 μg/mL, from the samples PCL and PCL/PEG was examined after 1, 2, 4, 6, and 24 h of incubation at 37 °C in PBS (Figure 5). To mitigate potential influences from PCL and/or PEG degradation during measurements, differences in absorbance between WAAPV-loaded and unloaded systems were considered (as reported in [8]). Because of the absence of inherent fluorescence in alanine, proline, and valine, the release of the AAPV could not be mapped (only tryptophan has intrinsic fluorescence [47]).

Fibers incorporating PEG reported larger degradation propensity due to their water affinity (Table 2 and Table 5). Yet, data from Figure 5 demonstrated that this propensity can be somewhat mitigated via the combination with the peptide, since both in the presence and absence of PEG, the peptide release profiles were very similar. WAAPV release was most important between the 2 h and the 6 h testing, with nearly 52% of the peptide being freed from the samples. After this period and up to 24 h testing, another 20% of peptide content was released. In the end, less than 33% and 22% of WAAPV remained on the PCL and PCL/PEG samples, respectively. In fact, it is estimated that concentrations of ≈ 33.5 and ≈ 38.8 μg/mL of the peptide were released from PCL/PEG + WAAPV and PCL + WAAPV fibers, respectively. Regardless of the PEG degradation profile, considering the hydrophobic interaction between PCL and WAAPV (both hydrophobic molecules), a superior release of the peptide in the presence of PEG was expected.

### 3.6. HNE Inhibition by Peptide-Loaded Electrospun Fibers

**Figure 6:**
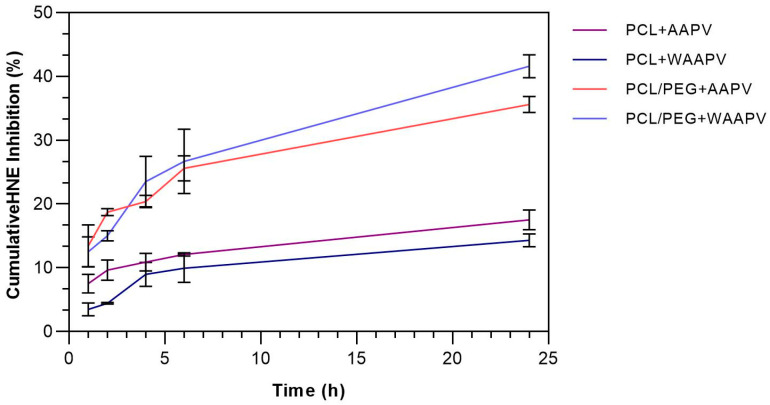
The HNE inhibition profile induced by AAPV- and WAAPV-loaded electrospun fibers over an incubation period of 24 h (*n* = 3).

AAPV- and WAAPV-loaded electrospun fibers were submerged in HNE-containing solutions and their ability to inhibit the enzyme activity after 1, 2, 4, 6, and 24 h of incubation in PBS at 37 °C was monitored (Figure 6). Data from peptide-unloaded fibers were also collected, and their interference subtracted from the peptide-containing fibers.

During the first 6 h of incubation, nearly 10% of HNE was inhibited by the peptide released from the PCL fibers. This is consistent with the data from Figure 5 since, at this point, >50% of the peptide molecules have been released. In fact, in the absence of PEG, HNE inhibition reached its maximum at this stage, maintaining the levels of enzyme activity in a constant manner until the 24 h period. By looking at the results from Table 1, it would be expected for WAAPV-induced HNE inhibition to be superior to that of AAPV. However, considering its hydrophobic nature and affinity towards PCL, it is likely for WAAPV release and access to HNE to be conditioned by this polymer, potentially by blocking the same binding sites used for HNE interactions, which may remain inactive even after separation from the microfibers. Data from AAPV activity against HNE, in contact with PCL scaffolds, are consistent with Miranda et al.’s results [8]. However, in contrast with the previous report, here, the activity of AAPV against HNE occurred in a quicker manner, reaching its maximum at 6 h instead of 24 h and maintaining its inhibitory activity as somewhat constant until the end of the experiment. This demonstrates, even if indirectly, that AAPV release kinetics from electrospun microfibers occurs at a faster rate than from within co-axial wet-spun microfibers because of the absence of a protective barrier.

Regarding the combination PCL/PEG, it was observed that >20% of the HNE is inhibited within the first 4 h of contact (the double of PCL fibers). It appears that peptide release and action are potentiated in the presence of PEG, probably in response to its affinity with water and accelerated degradation in comparison with PCL. However, after this point and until the 24 h contact, the increase in HNE inhibition continues, even above the inhibitory potential of the 50 μg/mL of the peptide (Table 1). According to Aimetti et al., as the substrate solution is converted and the enzyme inhibited, free carboxylic acids become available, turning the immersing solution containing fragments of degraded PEG into a polyelectrolyte. At this stage, the peptide molecules become more cationic and electrostatic attraction between them and PEG is potentiated [48], leading to a misreading of the HNE inhibition data. This also occurs because PEG is detected at 405 nm [49] and even though the influence of the polymers is excluded from the outcomes, these peptide–PEG complexes can only be accounted for when the peptides are loaded into the fibers.

### 3.7. Time-Kill Kinetics

**Figure 7:**
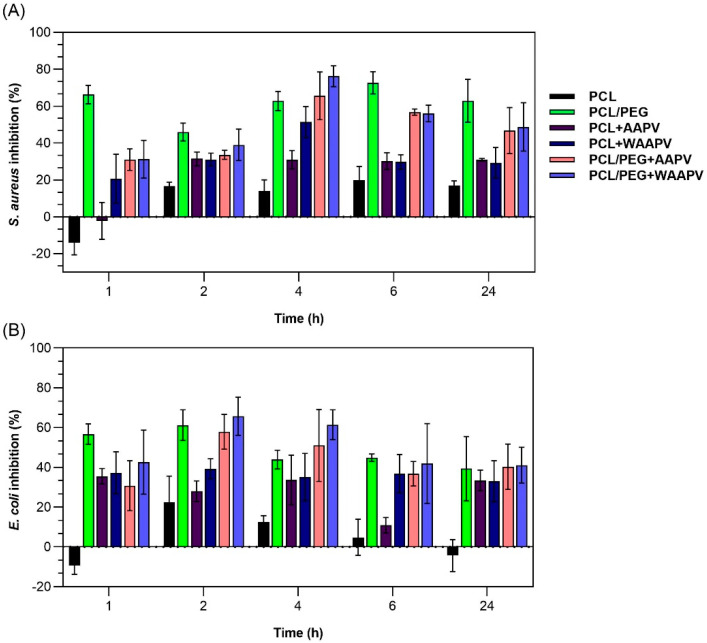
(**A**) *S. aureus* and (**B**) *E. coli* bacteria inhibition by unloaded and AAPV- or WAAPV-loaded electrospun fibers after 1, 2, 4, 6, and 24 h dynamic culture at 37 °C (*n* = 3).

Gram-positive (*S. aureus*) and Gram-negative (*E. coli*) bacteria growth inhibition induced by unloaded and peptide-loaded electrospun fibers was examined after 1, 2, 4, 6, and 24 h of incubation at 37 °C and a dynamic environment. Results were expressed in the percentage of inhibition as depicted in Figure 7.

From the very first moments of incubation, PEG-containing fibers were the ones reporting the highest bacterial inhibitions, a minimum of ≈30% against *S. aureus* and ≈40% against *E. coli*. Even though these values suggest an increased susceptibility against Gram-negative bacteria, growth inhibition was in most tested fibers more important against the Gram-positive bacteria (up to ≈78% and ≈66% against *S. aureus* and *E. coli*, respectively), thus confirming the observations made in Section 3.3. In general, the highest ratings for peptide-induced inhibition (in many instances potentiated by PEG) were reported at the 4 h mark against *S. aureus* and at the 2 h mark against *E. coli*, decreasing afterwards. According to Figure 5, at these time points, only ≈4 to ≈27% of the peptide WAAPV was liberated from the surfaces, hence suggesting that bacterial inhibition not only occurs through the penetration of a free peptide within the bacterial cells but also by contact with the peptide-loaded fibers. Most interestingly, by assessing the inhibition ratings promoted by fibers without PEG, it is evident that on their own, peptides also demonstrate antibacterial potentialities. Considering that this is the first report on the WAAPV capacities for bacterial inhibition, it is not possible to explain such an event, particularly considering the hydrophobic interactions established with PCL and the previous thermal and hydration data that suggested the peptide’s preferential location at the innermost region of the microfibers. Still, in a previous work conducted by our team, AAPV was also found to potentiate this inhibitory activity against *S. aureus* while loaded at the core of co-axial microfibers [8]. Even though more testing is required to take any definitive conclusions, it seems that peptide binding to polymer-based scaffolding systems induces a conformation and orientation that enables their interaction with bacteria. In the future, additional testing will be undertaken to corroborate these premises and explore in more detail the AAPV and WAAPV mechanisms of action while loaded and unloaded onto fibrous structures.

Overall, it was seen that the combination PCL/PEG + WAAPV was the one promoting the highest bacterial inhibition, regardless of bacteria type (Gram-positive or Gram-negative), by allying the repellent functions of PEG [30] to the uncovered (but still unclear) antibacterial features of WAAP.

## 4. Conclusions

In diabetic ulcer care, a combination of protection, exudate absorption, hydration capacity, bioactive agent-controlled release, proteolytic enzyme regulation, and inhibition of bacterial activity makes up the necessary ingredients for a successful treatment. In this study, multifunctional electrospun fibers were engineered with elastase-targeting peptides, AAPV and WAAPV. Characterization studies attested to the WAAPV abilities in regulating proteolytic enzyme action, by successfully inhibiting HNE. Microscale fibers with desirable wettability and controlled degradation profiles, which guaranteed peptide continuous release over a period of 24 h, were produced. Fiber loading of peptides was confirmed indirectly by thermal stability and wettability/hydration capacity studies. HNE was successfully inhibited by both AAPV and WAAPV. However, in the presence of PEG, the inhibition of HNE was highly increased, with PEG potentially interacting with the peptide molecules and generating complexes measurable at the same wavelength as the HNE detection kit. Peptide-loaded samples showcased antibacterial efficacy against both *S. aureus* and *E. coli*, achieving the highest ratings at the 4 h and 2 h marks, respectively. Future investigations should explore the fibers’ ability in inhibiting bacteria proliferation in chronic wound mimicking environments, considering factors such as pH and the presence of polymicrobial cultures. Additionally, exploring the potential benefits of the system towards cell regeneration will be crucial. As a first overview of the WAAPV potential for diabetic ulcer care, data can be deemed successful.

## Figures and Tables

**Table 1:** Inhibition of HNE activity by AAPV [8] and WAAPV at varying concentrations (*n* = 3).

Concentration (μg/mL)	HNE Inhibition (%)	
	AAPV	WAAPV
200.00	5.74 ± 0.010	11.94 ± 6.530
100.00	9.53 ± 0.010	12.22 ± 9.426
50.00	**16.89 ± 0.010**	**17.38 ± 5.441**
25.00	12.69 ± 0.002	9.66 ± 9.400
12.50	9.56 ± 0.011	11.68 ± 1.811
6.25	8.84 ± 0.002	14.25 ± 3.009
3.13	8.07 ± 0.007	15.36 ± 8.564
1.56	6.43 ± 0.005	21.91 ± 3.562
0.78	4.07 ± 0.004	13.36 ± 6.001
0.39	3.43 ± 0.005	9.09 ± 10.200
0.20	2.37 ± 0.007	10.20 ± 3.002
0.10	0.79 ± 0.008	7.92 ± 1.781

**Table 2:** Water contact angle determination (*n* = 6).

Samples	Water Contact Angle (°)	Image
PCL	131.43 ± 9.04	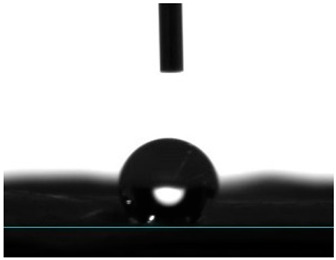
PCL/PEG	17.16 ± 3.19	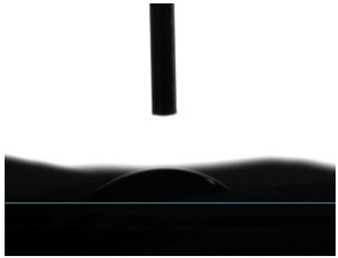
PCL + AAPV	109.55 ± 16.56	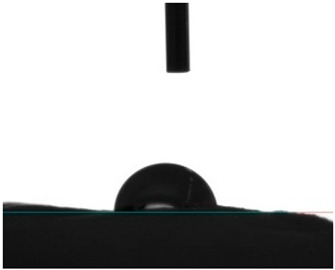
PCL + WAAPV	127.86 ± 6.08	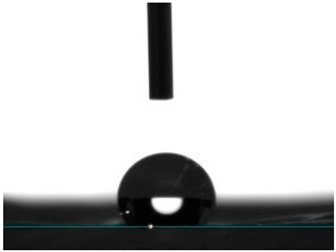
PCL/PEG + AAPV	0	-
PCL/PEG + WAAPV	0	-

**Table 3:** Electrospun mats’ porosity and air permeability rates (*n* = 3).

Samples	Porosity (%)	Air Permeability (L/min)
PCL	16.455 ± 0.066	0.523 ± 0.057
PCL/PEG	28.118 ± 1.001	1.878 ± 0.172
PCL + AAPV	15.012 ± 0.106	0.510 ± 0.018
PCL + WAAPV	17.588 ± 1.090	0.540 ± 0.105
PCL/PEG + AAPV	30.180 ± 1.255	1.997 ± 0.101
PCL/PEG + WAAPV	27.992 ± 1.880	1.769 ± 0.033

**Table 4:** DS (%) of electrospun fibers, unloaded and loaded with AAPV and WAAPV, immersed in PBS after 4, 6, 8, and 24 h of incubation at 37 °C (*n* = 3).

Samples	Degree of Swelling (%)			
	4 h	6 h	8 h	24 h
PCL	33.65 ± 0.22	22.08 ± 0.20	14.75 ± 0.48	12.11 ± 0.05
PCL/PEG	91.18 ± 1.62	85.83 ± 2.31	79.60 ± 1.50	78.08 ± 1.59
PCL + AAPV	41.05 ± 1.00	33.36 ± 0.02	25.06 ± 0.81	27.23 ± 1.22
PCL + WAAPV	28.05 ± 1.09	12.84 ± 1.06	22.75 ± 0.54	20.41 ± 0.65
PCL/PEG + AAPV	98.01 ± 0.24	95.00 ± 0.68	88.60 ± 0.45	80.11 ± 0.79
PCL/PEG + WAAPV	87.15 ± 1.09	80.45 ± 1.06	62.00 ± 1.26	59.17 ± 0.44

**Table 5:** Degradation profile of the unloaded and peptide-loaded electrospun fibers over 7 days of incubation in PBS at 37 °C and static testing conditions (*n* = 3).

Samples	Mass Loss (%)						
	Day 1	Day 2	Day 3	Day 4	Day 5	Day 6	Day 7
PCL	−5.03 ± 0.11	−26.17 ± 0.35	−47.10 ± 1.78	−62.21 ± 0.88	−56.07 ± 0.95	−67.12 ± 0.35	−55.50 ± 1.17
PCL/PEG	15.02 ± 0.35	9.98 ± 1.23	17.66 ± 1.64	18.02 ± 1.04	17.82 ± 1.20	17.95 ± 0.99	20.43 ± 0.28
PCL + AAPV	1.88 ± 0.02	−4.41 ± 0.23	−8.22 ± 1.00	−22.59 ± 1.02	−36.08 ± 1.60	−41.11 ± 0.55	−40.06 ± 0.77
PCL + WAAPV	−3.87 ± 0.88	−16.25 ± 0.87	−29.58 ± 1.07	−42.00 ± 1.58	−40.12 ± 0.33	−50.24 ± 0.69	−53.02 ± 0.94
PCL/PEG + AAPV	22.88 ± 0.16	19.00 ± 1.20	27.47 ± 0.48	28.11 ± 0.94	30.01 ± 1.55	29.05 ± 1.92	30.11 ± 0.97
PCL/PEG + WAAPV	18.12 ± 0.99	18.77 ± 0.03	19.51 ± 1.00	18.55 ± 0.58	22.12 ± 1.88	20.84 ± 0.67	20.00 ± 0.90

## Data Availability

Data is contained within the article.
